# Maternal predator odour exposure programs metabolic responses in adult offspring

**DOI:** 10.1038/s41598-018-26462-w

**Published:** 2018-05-24

**Authors:** Sophie St-Cyr, Sameera Abuaish, Kenneth C. Welch, Patrick O. McGowan

**Affiliations:** 1 Department of Biological Sciences, University of Toronto, Scarborough Campus, 1265 Military Trail, Toronto, ON Canada; 2 Center for Environmental Epigenetics and Development, Department of Cell and Systems Biology, University of Toronto, Toronto, ON Canada; 3 Department of Psychology, University of Toronto, Toronto, ON Canada; 4 Department of Physiology, University of Toronto, Toronto, ON Canada; 5 Present Address: The Children’s Hospital of Philadelphia, Colcket Translational Research Building, Department of Pathology and Laboratory Medicine, 3501 Civic Center Boulevard, Philadelphia, PA USA

## Abstract

A cardinal feature of the reaction to stress is the promotion of energy mobilization, enabling appropriate behavioural responses. Predator odours are naturalistic and ecologically relevant stressors present over evolutionary timescales. In this study, we asked whether maternal predator odour exposure could program long-term energy mobilization in C57BL/6 mice offspring. To test this hypothesis, we measured rates of oxygen consumption in prenatally predator odour exposed mice in adulthood while controlling for levels of locomotor activity at baseline and under stress. Circulating thyroid hormone levels and the transcript abundance of key regulators of the hypothalamic-pituitary-thyroid axis within the periventricular nucleus (PVN) of the hypothalamus and in the liver, including carriers and receptors and thyrotropin-releasing hormone, were measured as endocrine mediators facilitating energy availability. Prenatally predator odour exposed mice of both sexes mobilized more energy during lower energy demand periods of the day and under stressful conditions. Further, prenatally predator odour exposed mice displayed modifications of their hypothalamic-pituitary-thyroid axis through increased circulating thyroxine and thyroid hormone receptor α within the PVN and decreased transthyretin in the liver. Overall, these results suggest that maternal exposure to predator odour is sufficient to increase long-term energy mobilization in adult offspring.

## Introduction

A cardinal feature of the response to stress is the promotion of energy mobilization. This energy mobilization mediates the ‘fight-or-flight’ response, enabling behavioural responses to stress^1^. Long-term phenotypic changes are associated with stress-induced alterations in energetics. For example, chronic exposure to predators and predator cues in adult mice induces innate physiological responses such as a decrease in body weight and food intake, increased immobility, and increased heart and breathing rate^2^. Studies in the ecology literature have shown that exposures during perinatal life to environmental stressors present during the evolutionary diversification of species lead to adaptive programming of physiological systems involved in energy mobilization to match their predicted environment later in life^3^. Some of these physiological changes have been proposed to occur through alterations in hypothalamic-pituitary thyroid (HPT) axis and thyroid hormone function, involving increased circulating thyroid hormone levels as a key mediator of energy requirements in prenatally stressed animals^1,4,5^.

Developmental predator stress in amphibians, birds and fish has been shown to alter energy mobilization and demand through physiological, including metabolic, changes. Tadpoles developing in the presence of predators (larval dragonfly) exhibit reduced growth and deeper tails as a trade-off of growth for survival facilitating anti-predatory tactics^6^. In stickelback fish, eggs of gravid mothers exposed to predators contain higher concentrations of cortisol, and offspring show increased oxygen consumption in adulthood when compared to controls^7^. Song sparrows and zebra finches exposed to acute corticosterone supplementation mimicking an acute stressor during development, such as an encounter with a predator, display an increase in basal metabolic rate, especially at night during their low activity period^8,9^. In these studies, endocrine and gene expression mechanisms associated with these metabolic changes were not examined. To our knowledge, the only study to examine mechanisms by which metabolic alterations may occur with stress hormone exposure found that late fetal exposure to dexamethasone (a synthetic glucocorticoid) in rats leads to increased activity of key gluconeogenic enzymes such as glucose-6-phosphatase phosphoenolpyruvate carboxykinase within the liver, the organ responsible for energy mobilization^10^.

The HPT axis responds to increased energy demands with the release of thyrotropin-releasing hormone (*Trh*) from the hypothalamus that reaches the pituitary through portal circulation. The pituitary in turn releases thyroid-stimulating hormone (*Tsh*) into the general circulation that eventually reaches the thyroid gland and induces the release of thyroid hormones (thyroxine [T_4_] and triiodothyronine [T_3_]). T_4_ is catalyzed locally within target tissues to the active form T_3_ by iodothyronine deiodinase 2 (*Dio*2), which binds to the thyroid hormone receptors alpha and beta (*Thrα* and *Thrβ*). Thyroid hormones mediate an increase in core body temperature and energy consumption, in part through their action on the liver^4^. Hypothalamic-pituitary-adrenal (HPA) regulation of the endocrine stress response is under negative feedback inhibition in part through several effectors of the HPT axis, including *Tsh* and, and to a lesser extent, thyroid hormones themselves^11^. Circulating T_4_ in the blood is bound at 75% to liver-synthesized thyroxine-binding globulin (*Tbg*) and transthyretin (*Ttr*) and to albumin (15%)^11^.

We previously reported that maternal predator odour exposure in mice and rats alters HPA function in adult offspring, who show increased stress-related behaviors and corticosterone in the context of a stress challenge^12,13^.The objectives of the present study were to evaluate the impact of maternal predator odour exposure on indices of metabolic programming in male and female offspring by examining: (1) body weight and food consumption, (2) energy consumption (oxygen consumption) over 24 hours, (3) metabolic rate under acute stress (first exposure to a predator odour), and (4) the regulation of the HPT axis and its interaction with the HPA axis at baseline and following an acute stress (restraint) as a potential mediator of modifications in growth and energy consumption.

We exposed pregnant C57BL/6 mouse dams to predator odour during the second half of their pregnancy, the primary period of HPA axis development in offspring^14^. We hypothesized that offspring from predator odour-exposed dams (PO) would show long-term changes in metabolic responses, as evidenced by decreased body weight and food consumption and increased metabolic rate over 24 hours and under stress. We predicted that these changes would be associated with altered HPT function, including increased circulating T_4_ level and differential transcript abundance of genes regulating the HPT axis.

## Results

**Figure 1:**
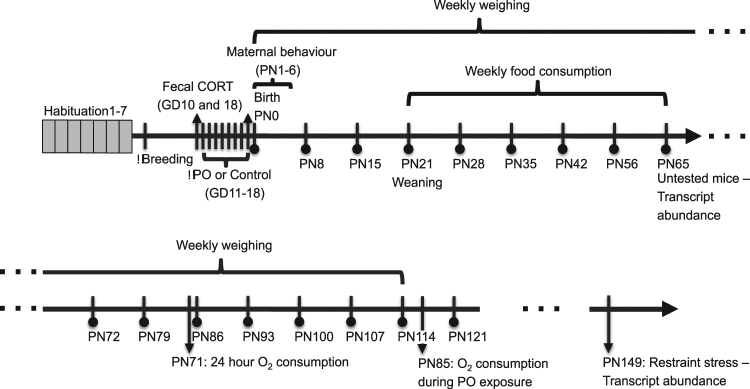
Experimental timeline. After a week of habituation to the odour exposure rooms, females were bred and exposed to control odour (distilled water) or randomized predator odour (bobcat or coyote urine or TMT, a component of fox feces) from GD 11–18. Maternal behaviour was measured on PN1–6. From birth to PN114, animals were weekly weighed. From PN21–65, the weekly food consumption was measured. On PN65, untested mice were sacrificed to measure basal transcript abundance. Adult mice O_2_ consumption over 24 hours was measured around PN85 and O_2_ consumption during predator odour exposure around PN119. Finally, a subset of tested mice was exposed to restraint stress around PN163 for stressed transcript abundance level. GD: Gestational Day; PN: Postnatal Day; PO: Predator Odour; TMT: 2,3,5-Trimethyl-3-thiazoline.

The timeline of the experiment is presented in Fig. 1.

### Fecal corticosterone metabolite levels during pregnancy

**Figure 2:**
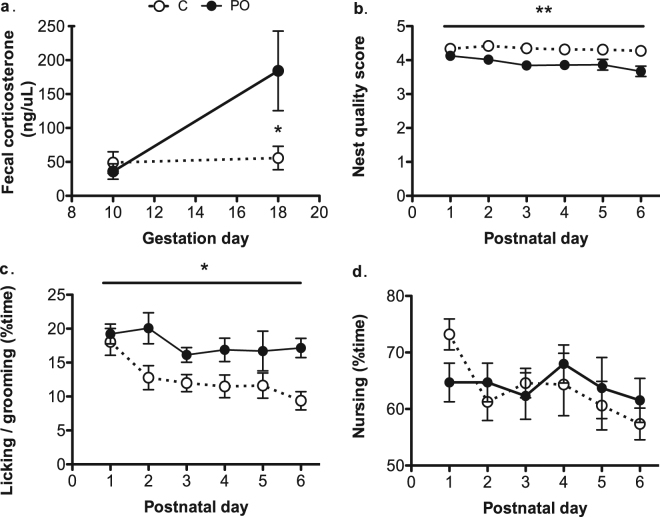
Increased fecal corticosterone metabolites during gestation and maternal behaviour alterations during the first postnatal week in predator odour (PO) exposed dams. Pregnant females showed increased fecal corticosterone metabolites after 8 days of PO exposure compared to control (C) pregnant females (**a**). PO dams showed lower nest quality (**b**) and more time-spent licking and grooming offspring (**c**) compared to control (C) dams. PO dams did not show alterations in nursing behaviour (**d**). Data are average ± standard error of the mean. Bars: PO exposure during pregnancy effect: *P ≤ 0.05; PO effect during pregnancy *P ≤ 0.05.

Predator odour was associated with increased fecal corticosterone metabolites in females exposed during pregnancy compared to unexposed controls [C: 58.775 ± 12.677, PO: 125.229 ± 37.773, *F*_*(1, 1*2*)*_ = 7.425, *P* = 0.02, *d*_*r*_ = 2.46; Fig. 2a]. As expected, there was no difference in corticosterone levels between the pregnant females at baseline prior to exposure (Gestational day [GD] 10) [*P* > 0.05] but PO female offspring exhibited higher fecal corticosterone metabolite on the last day of exposure [Mann–Whitney *U* = 7.40, *n*_C_ = 10, *n*_PO_ = 12, *P* = 0.05 two-tailed, *d* = 0.9; Fig. 2a].

### Maternal behaviours in dams and morphological measures in offspring

Predator odour-exposed dams built lower quality nests compared to control dams [C: 3.860 ± 0.051, PO: 4.316 ± 0.041, *F*_(*1*, 2*1*)_ = 8.9, *P* = 0.007, *pη*^*2*^ = 0.30] over postnatal days [PN] 1–6 (Fig. 2b). When controlling for the effect of nest quality, predator odour-exposed dams showed greater licking and grooming of their offspring relative to control dams [*F*_*(1, 132*)_ = 5.481, *P* = 0.02, *d*_*r*_ = 1.0; Fig. 2c]. Licking and grooming decreased in all dams over time [*F*_*(6, 152)*_ = 4.736, *P* = 0.001, *d*_*r*_ = 5.6]. There were no differences in the time spent nursing [*P* > 0.05; Fig. 2d], the length of pregnancy, pregnancy weight gain, litter size, offspring deaths up to weaning or litter sex ratios [*Ps* > 0.05].

**Figure 3:**
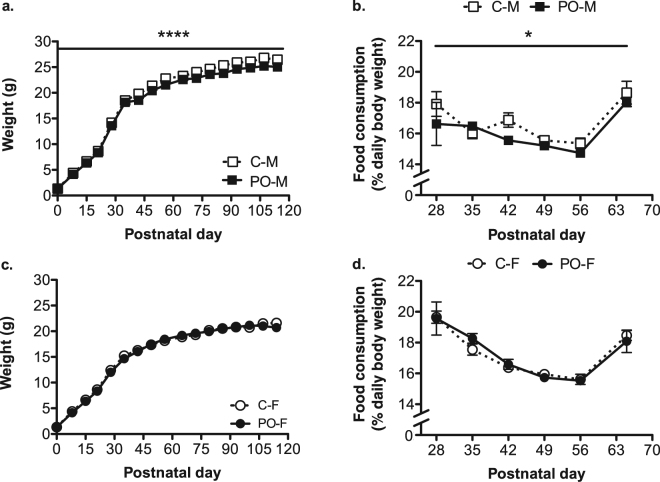
Decreased body weight and food consumption in male PO offspring. Male (M) PO offspring showed decreased body weight gain trajectory compared to male control (C) offspring (**a**). There was no difference between female (F) PO and control offspring body weight (**b**). Food consumption was comparatively lower overall in male PO offspring when compared to control males (**c**) whereas female PO offspring did not differ from controls (**d**). Data are average ± standard error of the mean. Angled bars: postnatal day effect: ****P ≤ 0.0001; Bars: PO effect: *P ≤ 0.05, ****P ≤ 0.0001.

The body weight was influenced by the PO and each sex differently [Interaction: *F*_*(1, 706)*_ = 9.515, *P* = 0.002, *d*_*r*_ = 6.5]. PO male offspring weighed less than control males overall [*F*_*(1, 343)*_ = 23.03, *P* < 0.0001, *d*_*r*_ = −0.8; Fig. 3a] while female offspring showed no PO effect on weight gain [*P* > 0.05; Fig. 3c]. Female offspring of both prenatal treatments showed no difference in age of sexual maturation [*P* > 0.05].

We measured food consumption after weaning, as an indicator of specific energy needs in offspring at different ages. The week after weaning, food consumption as a percentage of body weight decreased up to PN56 with a subsequent increase on PN65. PO male offspring showed a reduction in their food consumption as juveniles up to early adulthood compared to control males [*F*_*(1, 112)*_ = 5.472, *P* = 0.02, *d*_*r*_ = −0.5; Fig. 3b] and food consumption varied over time [*F*_*(5, 112)*_ = 7.429, *P* < 0.0001, *d*_*r*_ = 7.7]. Overall, food consumption by female offspring varied similarly over time [*F*_*(5, 116)*_ = 24.604, *P* < 0.0001, *d*_*r*_ = 4.7; Fig. 3d], but was not different in PO female offspring compared to control females [*P* > 0.05].

### Metabolic rate over 24 hours and during acute predator odour exposure in adult offspring

**Figure 4:**
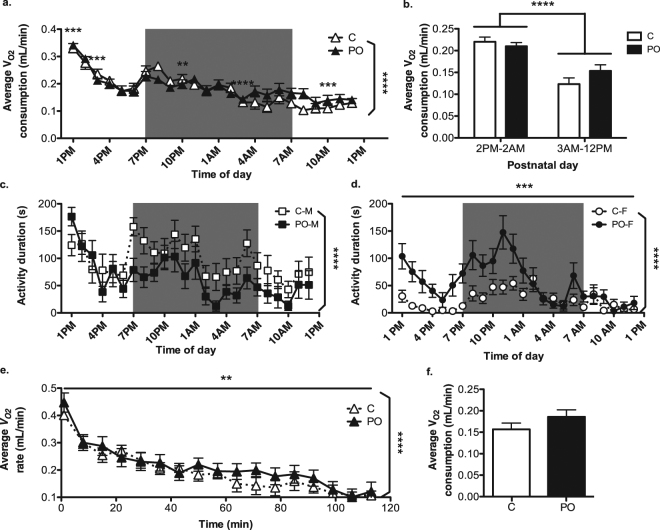
Differential average oxygen consumption rate over 24 hours and increased ${\dot{V}}_{{O}_{2}}$ during exposure to predator odour in PO offspring. Adult PO offspring showed differential average oxygen consumption $({\dot{V}}_{{O}_{2}})$ during 24 hours compared to control (C) offspring (**a**). There was an overall decrease in average ${\dot{V}}_{{O}_{2}}$ after 3AM in all mice (**b**). Male (M) PO offspring showed no difference in activity level variation over 24 hours compared to control males (**c**) while Female (F) PO offspring displayed increased overall activity when compared to control females (**d**). Data are average ± standard error of the mean. Shade: dark (active) phase of the light-dark cycle. Connected bar: time effect ****P ≤ 0.0001; Angled bar: time effect ****P ≤ 0.0001; Straight bar: PO main effect **P < 0.01, ***P ≤ 0.001; PO-exposure effect **P ≤ 0.01, ***P ≤ 0.001, ****P ≤ 0.0001.

The average oxygen consumption rate $({\dot{V}}_{{O}_{2}})$ over a 24 hr period differed over time in PO offspring compared to controls [Prenatal exposure x time interaction: *F*_*(23, 1004)*_ = 2.132, *P* = 0.001, *d*_*r*_ = 4.5; Fig. 4a]. PO offspring showed differential average ${\dot{V}}_{{O}_{2}}$ both during the light (1PM, 3PM and 10AM) and dark (10PM and 3AM) portions of the circadian cycle [*P* ≤ 0.002]. At the start of the trial (1PM) and from 3AM to 1PM the next day, PO offspring showed a higher average ${\dot{V}}_{{O}_{2}}$, while between 3PM and 1AM, PO offspring showed a decreased average ${\dot{V}}_{{O}_{2}}$. No significant sex effect was found regarding ${\dot{V}}_{{O}_{2}}$.

The lowest energetic needs in rodents, as indicated by minimal levels of circulating corticosterone, activity and metabolic markers, generally occurs between 3AM and 1PM of the light-dark cycle^15–17^. Accordingly, compared to the period between 3AM-12PM, ${\dot{V}}_{{O}_{2}}$ was higher between 2PM-2AM [*F*_*(2, 1051)*_ = 204.494, *P* < 0.0001, *dr* = −0.8; Fig. 4b].

Activity levels varied across the circadian cycle [*F*_*(23,1008)*_ = 8.902, *P* < 0.0001, *d*_*r*_ = 5.7], and were higher overall in males compared to females [*F*_*(1, 42)*_ = 30.838, *P* < 0.0001, *d*_*r*_ = −0.6]. Activity was also influenced differently in PO offspring of each sex [Interaction: *F*_*(1, 42)*_ = 13.988, *P* = 0.001, *d*_*r*_ = 2.8]. Males activity levels were not influenced by the prenatal exposure [*P* > 0.05; Fig. 4c], however PO females offspring showed increased activity overall compared to control females [*F*_*(1,20)*_ = 15.759, *P* = 0.001, *dr* = 0.7; Fig. 4d], an effect that varied over time [Interaction: *F*_*(23, 444)*_ = 2.147, *P* = 0.002, *d*_*r*_ = 13.7].

Overall, the quality of the nest built by adult offspring over the 24 hours during which ${\dot{V}}_{{O}_{2}}$ was measured was negatively correlated with average activity levels over the same period [*Pearson Correlation Coefficient (PCC)* = −0.36, N = 36, *P* = 0.03]. There was no difference overall between PO and control offspring in the quality of nests built during the period of metabolic measurement [*P* > 0.05].

The average ${\dot{V}}_{{O}_{2}}$ measured over the two-hours that mice were exposed to predator odour varied with time [*F*_*(16, 363)*_ = 16.633, *P* < 0.0001, *d*_*r*_ = 6.6] and was elevated overall in PO offspring [*F*_*(1, 366)*_ = 7.308, *P* = 0.007, *d*_*r*_ = 2.0] (Fig. 4e). When elevated ${\dot{V}}_{{O}_{2}}$ due to the initial stress of the metabolic chamber^18^ was excluded, the effect of PO remained significant [*F*_*(1, 334)*_ = 6.959, *P* = 0.009, *d*_*r*_ = 2.0]. No significant sex effect was found regarding ${\dot{V}}_{{O}_{2}}$.

The distance travelled in the metabolic chamber varied over time [*F*_*(16, 621)*_ = 9.501, *P* < 0.0001, *d*_*r*_ = 5.8]. The distance to the source of the predator odour also varied with time [*F*_*(16, 621)*_ = 15.426, *P* < 0.0001, *d*_*r*_ = 6.0] and between sexes [*F*_*(1, 37)*_ = 5,541, *P* = 0.02, *d*_*r*_ = 0.5] with females staying further away from the source of the predator odour.

### Circulating levels of thyroxine in adult offspring at baseline and in response to stress challenge

**Figure 5:**
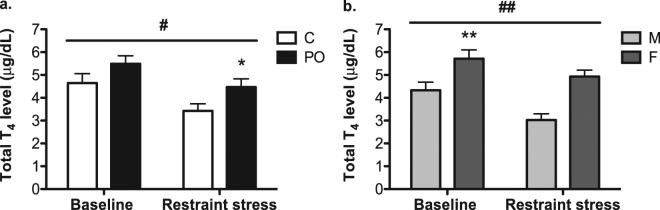
Elevated thyroxine levels in PO offspring. Adult PO offspring exhibited higher thyroxine (T_4_) level overall and after restraint stress compared to control (C) mice (**a**). Female offspring (F) had higher circulating T_4_ levels overall and at baseline compared to males offspring (M) (**b**). Data are average ± standard error of the mean. In **a**.: PO effect ^#^*P* ≤ 0.05; within restraint stress condition *P ≤ 0.05; in (**b**) sex effect ^##^*P* ≤ 0.01, within restraint stress condition **P ≤ 0.01.

PO mice offspring exhibited increased T_4_ levels overall (total level) compared to control mice [*F*_*(1, 72)*_ = 6.313, *P* = 0.02, *d*_*r*_ = 0.5; Fig. 5a]. Females offspring had higher T_4_ levels overall than males [*F*_*(1, 15)*_ = 8.740, *P* = 0.004, *d*_*r*_ = 1.1; Fig. 5b]. At baseline, circulating T_4_ levels were not influenced by PO-exposure [*Ps* > 0.05], while females had higher levels than males [*F*_*(1, 37)*_ = 7.882, *P* = 0.008, *pη*^*2*^ = 0.19]. After a restraint stress, PO offspring exhibited higher circulating T_4_ levels [*F*_*(1, 35)*_ = 6.182, *P* = 0.02, *pη*^*2*^ = 0.16]. Basal serum T_4_ levels in offspring were positively correlated with the amount of licking and grooming their mothers provided during the first postnatal week [*PCC* = 0.49, N = 32, *P* = 0.004].

### Transcript abundance of hypothalamic-pituitary thyroid axis genes

**Figure 6:**
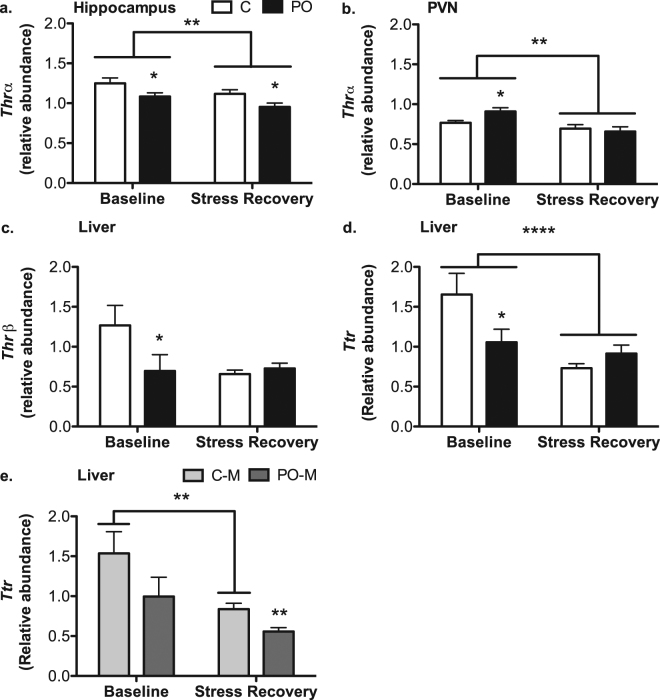
Altered transcript abundance of genes regulating the HPT axis in PO offspring. In the hippocampus, adult PO offspring exhibited lower thyroid hormone receptor alpha (*Thrα*) transcript abundance when compared to control (C) offspring (**a**). In the paraventricular nucleus of the hypothalamus (PVN), PO offspring compared to control offspring displayed elevated *Thrα* transcript abundance at baseline when compared to control mice (**b**). In the liver at baseline, PO offspring exhibited lower transcript abundance of thyroid hormone receptor beta (*Thrβ*; **c**) and transthyretin (*Ttr*; **d**), and lower abundance in male (M) PO offspring during stress recovery compared to control male mice (**e**). Hippocampal and PVN *Thrα* (**a**) and liver *Ttr* (**d**,**e**) showed lower transcript abundance overall during stress recovery when compared to baseline levels. Data are average ± standard error of the mean. Connected bars: stress effect **P ≤ 0.01, ****P < 0.0001; PO effect *P ≤ 0.05; **P ≤ 0.01.

**Figure 7:**
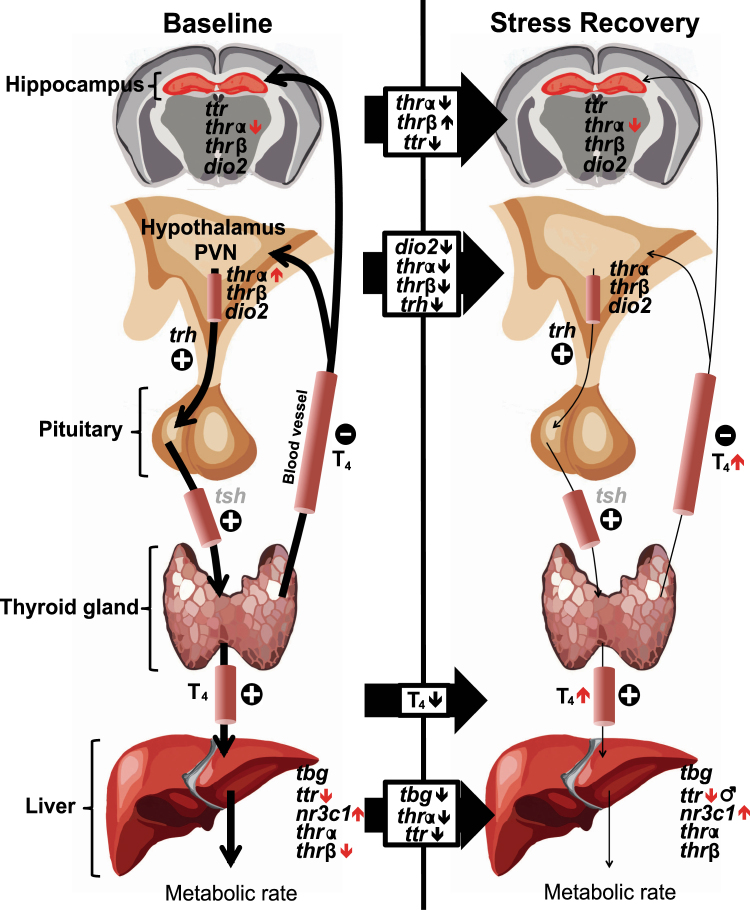
Modifications of the hypothalamic-pituitary thyroid (HPT) axis state at baseline and during stress recovery in PO offspring. Genes regulating the HPT axis that were examined in this study are shown. The black arrows indicate the direction of change in transcript abundance observed with stress challenge in both adult control and PO offspring. The red arrows indicate observed alterations in PO offspring. Overall, the changes in transcript abundance in PO offspring are consistent with increased HPT axis sensitivity and reactivity (see discussion). Genes investigated: *Ttr*: Transthyretin, *Thrα*: Thyroid Hormone Receptor Alpha, *Thrβ*: Thyroid Hormone Receptor Beta, *Dio2*: Iodothyronine Deiodinase 2, *Trh*: Thyrotropin-Releasing Hormone, T_4_: Thyroxine, *Tsh*: Thyrotropin-stimulating hormone, *Tbg*: Thyroxine-Binding Globulin, *Nr3c1*: Glucocorticoid receptor.

Based on our initial hypotheses, we structured our analysis of key genes within the HPT axis to examine the effect of stress challenge and PO. We also examined sex differences in the response to stress challenge and PO. Due to the number of analyses, primary effects are shown in Fig. 6, whereas Fig. 7 provides a schematic overview of our findings.

In the hippocampus, transcript abundance of *Thrα* was lower during restraint stress recovery compared to the baseline condition [*F*_*(1, 47)*_ = 6.936, *P* = 0.01, pη^2^ = 0.14; Fig. 6a]. In addition, *Ttr* transcript level was decreased and the *Thrβ* transcript abundance was increased during restraint stress recovery when compared to the baseline abundance [*Thrβ*: *F*_*(1, 47)*_ = 19.110, *P* < 0.0001, pη^2^ = 0.31; *Ttr*: *F*_*(1, 47)*_ = 70.561, *P* < 0.0001, pη^2^ = 0.63]. PO offspring showed lower *Thrα* transcript abundance compared to control mice in both stress conditions [Baseline: *F*_*(1, 23)*_ = 4.573, *P* = 0.05, pη^2^ = 0.19; Restraint stress recovery: *F*_*(1, 23)*_ = 5.494, *P* = 0.03, pη^2^ = 0.22; Fig. 6a]. Females had higher levels of *Thrα* than males [Male: 1.041 ± 0.036, Female: 1.161 ± 0.047, *F*_*(1, 47)*_ = 5.192, *P* = 0.03, pη^2^ = 0.11]. There was no effect of PO, stress challenge or sex differences in the expression of *Dio2* in the hippocampus.

In the PVN, *Thrα, Trh*, *Thrβ* and *Dio2* transcript abundance were reduced during restraint stress recovery compared to baseline levels [*Thrα*: *F*_*(1, 45)*_ = 11.030, *P* = 0.002, pη^2^ = 0.22 [Fig. 6b]; *Trh*: *F*_*(1, 46)*_ = 9.028, *P* = 0.005, pη^2^ = 0.18; *Thrβ*: *F*_*(1, 46)*_ = 9.679, *P* = 0.003, pη^2^ = 0.19; *Dio2*: *F*_*(1, 42)*_ = 17.233, *P* < 0.0001, pη^2^ = 0.32]. PO offspring showed an increase in *Thrα* at baseline [*F*_*(1, 23)*_ = 6.789, *P* = 0.02, pη^2^ = 0.25; Fig. 6b]. *Trh* did not exhibit a difference in PO offspring, however *Trh* transcript abundance was positively correlated with *Thrα* at baseline [*PCC* = 0.45, N = 24, *P* = 0.03]. No sex differences in transcript abundance of the genes examined were detected in the PVN.

In the liver, *Tbg, Thrα and Ttr*, transcript abundance decreased during restraint stress recovery as compared to baseline [*Tbg*: *F*_*(1, 62)*_ = 10.481, *P* = 0.002, pη^2^ = 0.16; *Thrα*: *F*_*(1, 62)*_ = 16.685, *P* < 0.0001, pη^2^ = 0.23; *Ttr: F*_*(1, 62)*_ = 16.423, *P* < 0.0001, pη^2^ = 0.22; Fig. 6c]. PO offspring had a lower liver *Ttr* transcript abundance at baseline [*F*_*(1, 20)*_ = 4.226, *P* = 0.05, pη^2^ = 0.17; Fig. 6c]. PO offspring showed lower *Thrβ* at baseline when compared to control mice [*F*_*(1, 23)*_ = 5.375, *P* = 0.03, pη^2^ = 0.21; Fig. 6d]. Overall, PO was associated with a higher transcript abundance of glucocorticoid receptor (*Nr3c1*) when compared to controls [*F*_*(1, 62)*_ = 4.628, *P* = 0.04, pη^2^ = 0.08; Fig. 6e]. Among male offspring, there was a significant interaction between PO and stress [*F*_*(1, 35)*_ = 9.984, *P* = 0.003, pη^2^ = 0.24]. Only control males showed decreased *Ttr* transcript abundance following restraint stress relative to baseline [*F*_*(1, 18)*_ = 13.091, *P* = 0.002, pη^2^ = 0.44; Fig. 6f] while among PO male offspring *Ttr* transcript abundance was similar across the two stress conditions [*P* > 0.05]. Male PO offspring had lower *Ttr* transcript abundance than control males [*F*_*(1, 23)*_ = 89.563, *P* = 0.008, pη^2^ = 0.28; Fig. 6f]. Males had higher *Thrβ* transcript abundance than females [*F*_*(1, 62)*_ = 13.461, *P* = 0.001, pη^2^ = 0.19; Restraint stress recovery: _38_57.128 < 062].

Food consumption during adolescence was positively correlated with restraint stress recovery *Ttr* transcript abundance in the liver [*Ttr*: *PCC* = −0.52, N = 30, *P* = 0.003]. Further, liver *Ttr* transcript abundance during stress recovery was correlated with body weight during adolescence and adulthood [Adolescence: *PCC* = 0.48, N = 31, *P* = 0.008; Adulthood: *PCC* = 0.49, N = 30, *P* = 0.006]. Finally, transcript abundance of *Thrβ* and *Ttr* at baseline in the liver was positively correlated with each other [*PCC* = 0.82, N = 23, *P* < 0.0001] suggesting that the hormone availability through the abundance of carrier may modulate the amount of hormone receptor.

## Discussion

We report here that maternal exposure to predator odour is associated with altered energy consumption in development and metabolic rate in adulthood in C57BL/6 mouse offspring. PO exposure was associated with a decrease in dams’ nest quality during the first postnatal week. Male PO offspring exhibited an overall decrease in body weight gain trajectory and comparatively lower food consumption. PO offspring showed an increased rate of oxygen consumption during the normally quiescent mid-day period and under acute stress upon a first exposure to a predator odour in adulthood. Increased circulating thyroid hormone levels and altered transcript abundance of key carriers and receptors regulating the HPT axis were observed within the hippocampus, PVN and liver. Overall, these results suggest that maternal exposure to predator odour was sufficient to increase long-term energy mobilization in adult offspring through alterations in HPT function.

### Maternal behaviour

Predator odour-exposure did not impact the amount of nursing or the total time spent on the nest by dams during the first postnatal week. However, dams exposed to predator odour during pregnancy showed increased postpartum licking and grooming behaviour, similar to reports of dams exposed during the first postnatal week to live predators or predator odour^19,20^. This effect on licking and grooming behaviour was detected despite the decreased nest quality observed in exposed dams. Lower nest quality decreases insulation, increases radiated heat and, subsequently, is known to increase litter oxygen consumption by as much as 25%, as nesting is a thermoregulatory behaviour^21^. Pups are poikilothermic until the third postnatal week, and are therefore susceptible to temperature variations during that time, especially males^22,23^. In previous studies, adult mice from a line selected for high quality nest building under low ambient temperatures showed greater basal metabolic rates, lower food consumption and higher body temperature, indicating that they may dedicate less of their energy intake to thermoregulatory demand^23,24^. Interestingly, pup licking and grooming have also been demonstrated to induce a modest but significant decrease in pup body temperature throughout the pre-weaning period^25^. Taken together, these results suggest that the behaviour of predator odour-exposed dams may alter pups’ thermoregulatory demand as a function of lower nest quality (insulation) and increased licking. A cross-fostering experiment should be conducted to clearly delineate the influence of prenatal and postnatal maternal influences, including maternal care, on the offspring phenotype. However, there is likely a strong prenatal contribution to the phenotype of PO offspring.

### Offspring physiology and metabolism

Several metabolic indices examined in offspring in this study appear to support our previous findings that maternal predator odour programs stress-related responses^12,13^. First, male PO offspring showed comparatively lower body weight from weaning to adulthood accompanied by lower food consumption during adolescence. Decreased body weight gain trajectories have been commonly observed following prenatal stress, including restraint stress^26,27^. Second, PO adult offspring in our study differed from controls in their rates of circadian variation of oxygen consumption, a proxy of the metabolic rate, independently of their body weight. PO offspring of both sexes showed increased ${\dot{V}}_{{O}_{2}}$ during the circadian period when the metabolic rates are known to be the lowest in mice (3AM-1PM) and were reduced during the period of the day normally showing the highest metabolic rates (2PM-2AM). The result was a flattening of the variation in ${\dot{V}}_{{O}_{2}}$ throughout the day in the PO offspring, more generally indicating a sustained ${\dot{V}}_{{O}_{2}}$, as occurs during stress exposure^2^. Third, female PO offspring, but not males, were hyperactive throughout the day. Increased activity is a common feature of the behavioural response to prenatal stress^28,29^. Activity levels in female offspring over a 24 hour period were correlated with the dam’s nest quality over the first postnatal week suggesting an association between adult activity and early life stress, as stress is associated with reduced nest building behavior^30^. However, unlike with their mothers, there was no evidence of a difference in the quality of nests that the PO mice offspring built over the 24 hours of the metabolic rate measurement. Therefore, variation in the quality of nest insulation does not appear to be responsible for the change in metabolic rate in adult offspring although it was correlated with the activity levels of the mice.

*Trh* and thyroid hormones are master regulators of feeding behaviour, thermogenesis and locomotor activation through weight loss and an increase in core body temperature, activity and energy expenditure^4^. A thyroid hormone-mediated energy expenditure increase is associated with an increase in oxygen consumption rate (up to 35% when compared to baseline) as early as the first postnatal week^31^.

We detected an overall increase in oxygen consumption rate when mice were exposed to predator odour for the first time postnatally, independent of their activity level or distance to the predator odour. Stress increases energy consumption and the mobilization of energy through the ‘fight-or-flight’ response. These results appear to be in accordance with the stress-induced hyperthermia detected in reaction to a wide range of stressors, which occurs together with increased energy mobilization and heart rate^32,33^. The lower nest quality observed in predator odour-exposed dams during the first postnatal week was negatively correlated with oxygen consumption rates during acute stress, further suggesting a biological association between maternal behaviour and later oxygen consumption in offspring subjected to acute stress.

These findings suggest that PO offspring exhibit long-term alterations in metabolism, with increased energy expenditure during the normally low energetic need period. High-energy consumption is related to a negative trade-off with growth, as we observed in PO male offspring. Furthermore, the resting metabolic rate is known to correlate with daily energy expenditure^34^ and, the resting metabolic rate has been correlated with behavioural traits (boldness), activity, productivity (growth, foraging activity) and ultimately fitness in a wide range of animal species^34,35^. It suggests that the physiological and metabolic changes in offspring discussed above (decreased body weight gain trajectory and food consumption in male PO offspring and an overall increase in oxygen consumption rate, especially under stress) are interrelated and influenced by maternal PO exposure. It is interesting to speculate that this integrative phenotype could in turn influence the global fitness of PO offspring, and be beneficial in an environment where predators are present, or detrimental in a non-stressful environment^36^.

### Offspring gene expression and thyroid hormone levels

The impacts of PO and stress exposure on modifications in transcript abundance in the HPT axis and circulating levels of T_4_ reported in this study are summarized in Fig. 7. Overall, restraint stress recovery (2 hours post stress-onset), was associated with decreased expression of several genes in the HPT axis within the PVN (*Dio2*, *Thrα*, *Thrβ* and *Trh*), liver (*Tbg*, *Thrα* and *Ttr*) and hippocampus (*Thrα* and *Ttr*). Interactions between the HPT axis and the HPA axis potentially mediated through *Trh* PVN neurons that receive *Crf* inputs, stimulating *Trh* biosynthesis^37^. Recent work by our group indicates that *Crf* transcript abundance is increased in the PVN of adult PO offspring (St-Cyr *et al*., *in preparation*), however no significant increase in *Trh* transcript abundance was detected in PO mice offspring. It appears more likely that increased *Thrα* abundance in the PVN may mediate increased T_4_ levels in PO offspring and sustained HPT axis activation^38^.

Elevated T_4_ levels could also participate in the stress-related phenotype reported in PO offspring^12,13^. Several studies have shown that elevated T_4_ levels induce an increase in corticosterone-releasing factor (*Crf*) and corticosterone levels^39–42^. Increases in the abundance of *Trh* and thyroid hormones receptors in the PVN in turn increase plasmatic levels of T_4_ while decreases in abundance of the carriers, *Tbg* and *Ttr*, in the liver increase plasmatic T_4_ availability. *Ttr* is a protein carrier of thyroid hormones that is synthesized in the liver and brain^43,44^ and is likely stress-responsive as its genomic sequence contains a conserved glucocorticoid response element^45^, with *Nr3c1* binding decreasing *Ttr* abundance^46^. *Ttr* therefore constitutes a potential target of early life programming since its transcript abundance is modulated by stress, as observed in several targets of prenatal stress (e.g. *Bdnf*, *Fkbp5*)^12,13^. Accordingly, we detected decreased *Ttr* transcript abundance in the liver following restraint stress. *Ttr* has also been linked to differences in predation stress^47^, making it a possible target for early-life predation cue exposure. Accordingly, PO offspring showed lower liver *Ttr* transcript abundance at baseline and higher liver *Nr3c1* transcript abundance at baseline and after stress. Typically, high-intensity stressors such as immobilization stress and corticosterone injections lead to an elevation of circulating thyroid hormones^48,49^. On the contrary, other stressors (such as a lipopolysaccharide injection or inescapable tail shock) suppress *Trh* transcript abundance^50^ or suppress thyroid hormone levels^51^, suggesting stress-specific effects.

PO adult mice showed a hippocampal-specific decrease in *Thrα*. Similarly, prenatal ethanol exposure in rats led to a reduction in the fetal hippocampal *Thrα* transcript abundance on the last day of pregnancy^52^. Increased sensitivity to thyroid hormone is found in *Thrα* KO mice^53^, suggesting that PO offspring may show enhanced sensitivity and reactivity to thyroid hormones. In accordance with these results, the hippocampus likely has a facilitating effect on the HPT axis through increased total T_4_ and free T_4_ levels, as observed in association with hippocampal lesions^54^.

### Sex differences

The only female-specific effect in PO offspring detected in this study was hyperactivity during the circadian rhythm. Females are normally more active than males but this difference was stronger in female PO offspring while their body weight and food consumption were similar to control females. These results are consistent with the similar body weights of female PO and control offspring reported previously by our group^12,13^.

During restraint stress recovery, *Ttr* showed decrease transcript abundance in the liver in male but not female PO offspring. Males have previously also been demonstrated to be more sensitive than females to changes in circulating levels of thyroid hormone in several brain regions, including the hippocampus^38^, likely through the lower levels of thyroid hormone receptor present in males. The lower weight gain trajectory and food consumption in PO male offspring was positively correlated with the transcript abundance of *Ttr* in the liver during stress recovery. This correlation suggests a potentially biologically relevant influence of stress and the HPT axis on food consumption and weight trajectories in males, potentially through the redistribution of the tissue-specific energy allocation.

In conclusion, these findings provide evidence that maternal PO exposure induces enduring metabolic adaptation in offspring. The oxygen consumption rate and transcript abundance of genes regulating the HPT axis were altered in the adult offspring of PO exposed dams. The observed increased circulating thyroid hormone levels may be a key mediator of energy requirements in PO offspring. However, the hypothalamic increase in thyroid hormone receptor abundance could buffer, at least in part, some aspects of the stress-related PO phenotype. Male PO offspring also showed more pronounced physiological modifications and changes in liver *Ttr* transcript abundance. However, both male and female offspring appear to have a more sensitive and reactive HPT axis as a function of decreased receptor and carrier abundance in the hippocampus and liver. In turn, this increase HPT axis sensitivity could lead to an increase in energy availability in PO offspring.

## Material and Methods

### Mice breeding

C57BL/6 mice were obtained from Charles River Canada (St. Constant, QC) and housed as reported previously^13^. For breeding, two normally cycling females were housed with one male overnight. Pregnant females were singly housed on GD 10 and weighed throughout pregnancy. Twenty-four of the 41 mated females (58.5%) gave birth to 12 control litters and 12 prenatally predator odour exposed litters. All experimental protocols were approved and conformed with guidelines and best practices established by the Local Animal Care Committee at the University of Toronto in Scarborough and in accordance with guidelines set forth by the Canadian Council on Animal Care.

### Fecal corticosterone metabolite levels during pregnancy

Feces were collected from pregnant females for corticosterone metabolites measurements. Females were housed in an empty cage from 9 to 10 PM for 7 days for habituation prior to mating. Feces were collected during the one hour, 6 hours after the stress exposure onset (4PM), reflecting the circulating corticosterone present during the stress^55^. Baseline levels were measured in pregnant females on GD10 and were compared to GD18. A subset of experimental females, 11 control and 13 predator-odour exposed pregnant females, were compared.

Fecal corticosterone metabolite extraction was conducted by drying the frozen feces overnight using a lyophilizer (Labconco), crushing them in liquid nitrogen, weighing (94 g on average), extraction in ethanol and dilution in methanol (1:40)^56,57^. The amount of corticosterone metabolites in the fecal extracts was determined using a radioimmunoassay kit with ^125^I-labeled anti-corticosterone antibody (MP Biomedicals Inc., CA., USA: sensitivity 7.7 ng/mL, intra-assay coefficient of variation 10.3%; cross-reactivity: corticosterone 100%, desoxycorticosterone 0.34%, cortisol 0.05%, aldosterone 0.03%, cholesterol/11-desoxycortisol <0.01%).

### Predator odour exposure in pregnancy and maternal behaviour in dams

The protocol for exposing pregnant dams to predator odour was described previously by our group^13^. Briefly, females were habituated to the hood and the odour exposure rooms and cages (15 × 33 cm) during the week prior to mating. From GD11 to 18, dams were exposed to liquid odours on cotton balls sealed in a perforated petri dish to avoid direct contact with the odorants. Dams in the predator odour exposure group were exposed once a day for 1 hour during the light cycle to an odour for a total of three times to 3 mL of bobcat urine, three times to 5 mL of coyote urine (thepeemart.com) and twice to 5 μL of a 1:5000 solution of 2,3,5-Trimethyl-3-thiazoline (TMT, a purified component of fox feces, Contech enterprises #300000368) in a randomized manner. Control dams were exposed to the same regimen at 4PM each day except that distilled water was used instead of the odorants. Maternal behaviours of predator odour exposed and control dams were examined on postnatal days (PN) 1–6 according to a previously published protocol^13^. On PN0, litters were culled to a maximum of 6 pups (litter sizes 3–6 pups). Nest quality was evaluated at the beginning of each observation according to a qualitative 1 to 5 scale, with 1 being scattered nesting material and 5 a closed dome-shaped nest^58^.

### Offspring physiological measures

Sex and body weight of pups (nearest 0.1 g) were measured on PN0. Offspring were left undisturbed except for a weekly weighing until PN21, when they were housed in same-sex and same prenatal treatment groups (3 to 5 per cage). Body weight measurements continued weekly up to PN114. Additionally, from PN 21–65, offspring food consumption was measured weekly.

### General testing procedures in adult offspring

Beginning at PN71, metabolic rates and activity in adult offspring (1–2/sex/litter) were examined in the following order: metabolic rate and activity over 24 hours (control male [C-M] N = 13, control female [C-F] N = 12, PO males [PO-M] N = 13, PO females [PO-F] N = 12), followed at least a week later by metabolic rate and activity during the first postnatal exposure to a predator odour (C-M N = 12, C-F N = 11, PO-M N = 12, PO-F N = 9). T_4_ levels at baseline were measured in a separate group of unstressed (C-M N = 10, C-F N = 11, PO-M N = 9, PO-F N = 10) and restrained mice (C-M N = 10, C-F N = 11, PO-M N = 9, PO-F N = 10). Gene transcript abundance was measured in a subset of untested mice and in a separate subset during recovery from a restraint stress (2 hours post-stress onset) (N = 6 per PO and control group, 1 mouse/sex/litter). Before any experiment, the mice were handled (3 minutes/day) for seven consecutive days prior to testing to habituate them to the experimenter. The order of testing for each experiment was pseudo-randomized between PO and control groups and sexes, with at least a week time interval between each test.

### Metabolic rate over 24 hours in adult offspring

The oxygen consumption rate $({\dot{V}}_{{O}_{2}})$, a proxy of the metabolic rate, of adult control and PO offspring was measured over a 24-hour period using open-flow respirometry. Mice were individually housed in Sable Systems mouse-sized respirometry cages (Model: CAGE-3721; Las Vegas, NV, USA) with polysulfone tubes fitted on a filter top and a respirometer manifold (Model: MAN-3721) permitting air intake. Up to 7 mice were recorded in turn over a 45-minutes period on each of the 24-hour recorded days. Eight respirometer cages were sequentially serially sampled with one of these cages left empty to provide a reference ambient air recording. Cages were provisioned with food pellets and water. Air was drawn through the cages at 800 mL/minute and flow rate was regulated by a Flowbar-8 flow controller system (Sable Systems). An RM-8 Flow Multiplexer, controlled by Expedata data collection software (V. 1.7.2; Sable Systems) orchestrated the sequential sampling: 5-minute reference chamber, animal chambers 1–4 5-minutes each, 5-minutes reference chamber, animal chambers 5–7, 5-minutes each. This sampling regime was repeated over the full 24-hour measurement period for each mouse.

Excurrent air from the focal respirometry chamber was drawn through the main pump (and flow meter/controller) of a Turbofox 5 respirometry system (Sable Systems). The airstream was then sub-sampled at a flow rate of approximately 200 mL/minute and this gas passed through the Turbofox’s water vapour meter, the CO_2_ analyzer and O_2_ analyzer. Analog signals from flow meters and gas analyzers were converted to digital signals via the internal A/D converter of the Turbofox. Data were recorded every 5 seconds to computer via Expedata software. The flow-rate from the focal chamber, and O_2_ and CO_2_ partial pressures were corrected for water vapour dilution using equation 8.6 in Lighton^59^. Drift in the O_2_ trace was corrected within the Expedata software suite by fitting a spline function through reference chamber recordings. ${\dot{V}}_{{O}_{2}}$ (in mL/minute) was calculated using equation 11.7 in Lighton^59^. Mice were filmed throughout testing and their activity level (walking/lateral movements) were blindly coded throughout the 5-minute metabolic rate recordings using Observer XT 8.5. The average ${\dot{V}}_{{O}_{2}}$ and activity from the recorded time bins were used for analysis.

### Metabolic rate during predator odour exposure in adult offspring

The ${\dot{V}}_{{O}_{2}}$ of adult control and PO mice offspring was measured during a two-hour exposure to an inescapable predator odour (100 μL TMT 1:5000). The respirometric setup and analyses were conducted identically to section **4.6** except that only 2 cages were used; one with a focal mouse and one left empty (reference). Cages were placed inside a fume hood to contain the predator odour. Therefore, the flow-rate through the cages was maintained at ~2000 mL/minute. Date from the reference chamber was recorded for 2 minutes and the animal metabolic chamber for 8 minutes. This schedule was repeated over 2 hours. Activity level was tracked continuously using EthoVision XT 10. The average ${\dot{V}}_{{O}_{2}}$ and activity from the recorded time bins were used for analysis.

A user error resulted in a needle valve moderating airflow through the Turbofox being left open during a subset of recordings. Because of this, the flow controller of the Turbofox was unable to maintain a steady flow rate and flow through the chambers fluctuated wildly. Calculation of accurate ${\dot{V}}_{{O}_{2}}$ could not be accomplished on this subset of data (11 mice), which were excluded from further analyses. The average ${\dot{V}}_{{O}_{2}}$ and activity from the recorded time bins were used for analysis. Further analysis was conducted while excluding the initial 8 minutes of elevated ${\dot{V}}_{{O}_{2}}$ as a result of the initial stress of the metabolic chamber^18^.

### Thyroxine in circulation at baseline and during recovery from restraint stress in adult offspring

Baseline and stress recovery T_4_ levels were measured in unstressed mice and, in a separate group, during recovery from restraint stress, respectively. On the day of testing, mice were habituated to the procedure room for two hours. Mice were then restrained in a transparent cone bag (Wilton) for 60 minutes and returned to their home cage. Restrained mice were sacrificed two hours after the stress onset and trunk blood was collected. Blood was kept on ice at least 30 min before being centrifuged at 4,000 rpm at 4 °C for 20 minutes. Serum was then extracted and stored at −80 °C. The amount of T_4_ in duplicate serum samples was determined using a commercially available RIA kit with ^125^I-labeled thyroxine antibody (MP Biomedicals Inc., CA., USA: thyroxine sensitivity 0.76 μg/dL, intra-assay coefficient of variation 11.4%).

### Transcript abundance analysis by quantitative real time-polymerase chain reaction in adult offspring

Transcript abundance in the hippocampus, PVN and liver were quantified in mice as described previously^13^. The genes examined included: *Ttr*, *Thrα*, *Thrβ* and *Dio2* in the hippocampus, *Thrα*, *Thrβ*, *Dio2* and *Trh* in the PVN, and *Ttr*, *Thrα*, *Thrβ*, *Tbg*, and *Nr3c1* in the liver. Transcript abundances were corrected using the housekeeping gene *Ywhaz* in the hippocampus^12,13^, the geometric mean of 4 housekeeping genes (*18s*, *Actinb*, *Gapdh* and *Ywhaz*) in the PVN and *18s* in the liver based on the stability of housekeeping genes in specific tissues^60^. Primers sequences are available in Supplementary Table S1.

### Statistical analysis

Statistical analyses were carried out using SPSS (IBM). Data were tested for normality using the Shapiro-Wilk test and were log- or square root-transformed to achieve normality whenever necessary. General linear models (GLM) or linear mixed models (LMM), to correct for litter effects or missing data, were used for analyses. When the data could not be normalized, a Mann-Whitney test was performed. Datasets over 30 samples (maternal behaviour, body weight) per group that could not be normalized were treated as normal distributions, as violations of the normality assumption with large samples sizes is not considered problematic^61^. Effect sizes were calculated using the Cohen’s d or d_r_ (calculated with the residuals of the LMM), with d ≥ 0.5 indicating moderate and d ≥ 0.8 indicating large effect sizes, or with eta squared (η^2^) or partial eta-squared (pη^2^), with η^2^ or pη^2^ ≥ 0.06 indicating moderate and η^2^ or pη^2^ ≥ 0.14 indicating large effect size^62,63^. Partial least-squares difference tests or Bonferroni corrected t-tests with GLM, or LMM respectively separated by sex were used for *post hoc* comparisons. Effects were considered statistically significant at *P* ≤ 0.05. Pearson correlations, for normal distributions, were used to assess the relationship between key variables. Additional details are available in Supplementary information.

### Data availability

The datasets generated during and/or analyzed during the current study are available from the corresponding author on reasonable request.

## Electronic supplementary material

Supplementary information
