# Supplementary material for: Maternal predator odour exposure programs metabolic responses in adult offspring
Source: Sci Rep. 2018 May 24;8:8077. doi: 10.1038/s41598-018-26462-w (PMC5967341; doi:10.1038/s41598-018-26462-w)
Supplement: Supplementary file 1 — Supplementary information [file 41598_2018_26462_MOESM1_ESM.pdf]

Supplementary information

Maternal predator odour exposure programs metabolic responses in adult offspring

Sophie St-Cyr, Sameera Abuaish, Kenneth C. Welch Jr. and Patrick O. McGowan

Additional measures showing similarities between PO and control offspring:

Maternal behaviours in dams and morphological measures in offspring

No significant difference in the time spent nursing ($F_{(1,21)}$ = 0.003, P = 0.96), the length of pregnancy ($F_{(1,22)}$ = 0.155, P = 0.698), the pregnancy weight gain ($F_{(1,22)}$ = 0.74, P = 0.399), litter size ($F_{(1,22)}$ = 0.218, P = 0.645), offspring death up to weaning (*Mann-Whitney U* = 105.00, n_c = 15, n_{PO} = 15, P = 0.748 two-tailed) or litter sex ratio ($t_{(1,22)}$ = 1.159, P = 0.259) were detected between control and PO dams. Age of sexual maturation was not significantly different between female PO and control offspring ($t_{(1,20)}$ = 0.362, P = 0.721).

Transcript abundance of hypothalamic-pituitary thyroid axis genes

Within the hippocampus, *Dio2* transcript abundance were not modified following a restraint stress ($F_{(1,42)}$ = 0.197, P = 0.660). Within the same tissue, *Thrβ*, *Dio2* and *Ttr* transcript abundance were not significantly different between control and PO offspring (*Thrβ* baseline: $F_{(1,20)}$ = 0.013, P = 0.909; *Thrβ* stress recovery: $F_{(1,15)}$ = 0.001, P = 0.981; *Dio2* overall: $F_{(1,42)}$ = 0.019, P = 0.891; *Ttr* baseline: $F_{(1,20)}$ = 0.320, P = 0.578, *Ttr* stress recovery: $F_{(1,20)}$ = 0.038, P = 0.848).

Within the PVN, *Thrβ*, *Dio2* and *Trh* transcript abundance were not significantly different between control and PO offspring (*Thrβ* baseline: $F_{(1,15)}$ = 0.916, P = 0.354; *Thrβ* stress recovery: $F_{(1,12)}$ = 0.812, P = 0.385; *Dio2* baseline: $F_{(1,21)}$ = 0.817, P = 0.378; *Dio2* stress recovery: $F_{(1,20)}$ = 0.891, P = 0.358; *Trh* baseline: $F_{(1,20)}$ = 0.503, P = 0.487; *Trh* stress recovery: $F_{(1,19)}$ = 0.250, P = 0.623).

Within the liver, *Nr3c1* and *Thrβ* transcript abundance were not modified following a restraint stress (*Nr3c1*: $F_{(1,57)} = 0.491$, $P = 0.486$; *Thrβ*: $F_{(1,56)} = 1.283$, $P = 0.262$). Within the same tissue, *Thrα* and *Tbg* transcript abundance were not significantly different between control and PO offspring (*Thrα* baseline: $F_{(1,21)} = 2.394$, $P = 0.139$, *Thrα* stress recovery: $F_{(1,38)} = 0.094$, $P = 0.761$; *Tbg* baseline: $F_{(1,23)} = 3.733$, $P = 0.068$; Tbg stress recovery: $F_{(1,38)} = 0.293$, $P = 0.592$).

Material and methods

Statistical analysis

Pregnant fecal corticosterone levels comparisons were analyzed using a LMM with predator odour exposure and gestational day as main factors and feces weight as a random factor. Within gestational day comparisons were performed using Bonferroni corrected Mann-Whitney Tests. Data for maternal behaviours were analyzed using 2(predator odour exposure) x 6(postnatal day) repeated GLM with litter size and nest quality as covariates. Data for body weight were analysed using a 2(prenatal treatment) x 17(weighing day) or 6(food consumption day) LMM while correcting for the size of the litter as a random factor.

A LMM with time, light phase or pre- and post- 3AM time as repeated measures and PO-exposure and sex as main effects was used to compare the average $\dot{V}_{O_2}$ over 24 hours and activity level while correcting for random factors such as body weight, distance travelled and litter ID. A LMM with time as a repeated measure and PO-exposure and sex as main effects was used to compare the average $\dot{V}_{O_2}$ during predator odour exposure while correcting for random factors (body weight, distance travelled, distance to the predator odour and litter ID). Hour-by-hour comparison were performed using Bonferroni corrected Mann-Whitney Tests.

T_4 level comparisons were analyzed using a LMM with PO-exposure, sex and presence of restraint stress as main factors with litter ID as a random factor.

Two(maternal predator odour exposure) x 2(sex) GLM or LMM followed by post-hoc analyses were used for baseline and restraint stress recovery transcript abundance analysis with litter ID as a random effect.

Correlational analysis was used to assess the relationship between key variables (licking-grooming over the first postnatal week, weight in adolescence and adulthood, food consumption during adolescence, activity level over 24 hours, nest quality during the first postnatal week and over 24 hours in adulthood, $\dot{V}_{O_2}$ during predator odour exposure, baseline T_4 serum level, PVN *thrα* and *trh* baseline transcript abundance, liver *ttr* and *thrβ* baseline transcript abundance, liver *ttr* stress recovery transcript abundance) influenced by prenatal predator odour exposure as functional and mechanistic connections were expected between those variables. Effects were considered statistically significant at $P \leq 0.05$. Pearson correlations, for normal distributions, were used to assess the relationship between variables.

Supplementary Table S1. Mice primer sequences

Gene	Forward primer (5′-3′)	Reverse primer (5′-3′)
18s	CCCTGAGAAGTTCCAGCACA	GTGATCACTCGCTCCACCTC
Actinb	TTTGAGACCTTCAACACCCC	ATAGCTCTTCTCCAGGGAGG
Dio2	TGTCTGGAACAGCTTCCTCCT	CTCTGCACTGGCAAAGTCAAG
Gapdh	CCTGCACCACCAACTGCTTA	CGTTCAGCTCTGGGATGACC
Nr3c1	AACTGGAATAGGTGCCAAGG	GAGGAGAACTCACATCTGGT
Tbg	TGGGCATGTGCTATCATCTTCA	GAGTGGCATTTTGTTGGGGC
Thrα	CAGGCTGTGCTGCTAATGTC	CTGCCCCCTTGTACAGAATC
Thrβ	CCTGGAAGCCTTCAGTCATT	AGTCTCGCTGTCTGGGTCAT
Trh	GCTGTGACTCCTGACCTTCC	ATCTCCCCTCTCTTCGGCTT
Ttr	GGACACCAAATCGTACTGGA	CAGAGTCGTTGGCCTGTGAAA
Ywhaz	TTGAGCAGAAGACGGAAGGT	GAAGCATTGGGGATCAAGAA

18s: 18S Ribosomal RNA; *Actinb*: Actin Beta; *Dio2*: Iodothyronine Deiodinase 2, *Gapdh*: Glyceraldehyde 3-Phosphate Dehydrogenase; *Nr3c1*: Glucocorticoid receptor, *Tbg*: Thyroxine-binding globulin; *Thrα*: Thyroid Hormone Receptor Alpha, *Thrβ*: Thyroid Hormone Receptor Beta, *Trh*: Thyrotropin-releasing hormone, *Ttr*: Transthyretin; *Ywhaz*: 14-3-3 protein zeta/delta.
